# A comparative analysis of stem cells derived from young rabbit knee joints: the potentially superior performance of decellularized extracellular matrix pretreated infrapatellar fat pad stem cells on nanofiber scaffolds

**DOI:** 10.3389/fcell.2025.1539308

**Published:** 2025-03-26

**Authors:** Zhixin Wei, Qingqing Yu, Qingyun Xie, Dongfa Liao, Xue Gou, Song Chen

**Affiliations:** 1 College of Medicine, Southwest Jiaotong University, Chengdu, Sichuan, China; 2 Department of Orthopaedics, The General Hospital of Western Theater Command, College of Medicine, Southwest Jiaotong University, Chengdu, Sichuan, China; 3 Institute of Biomedical Engineering, College of Medicine, Southwest Jiaotong University, Chengdu, China; 4 Pancreatic Injury and Repair Key Laboratory of Sichuan Province, The General Hospital of Western Theater Command, Chengdu, China

**Keywords:** mesenchymal stem cells, decellularized extracellular matrix (dECM), electrospun nanofiber scaffolds, osteogenesis, adipogenesis, chondrogenesis

## Abstract

**Introduction:**

Osteoarthritis (OA) remains a significant clinical challenge, necessitating improved strategies for cartilage repair. Stem cells and scaffolds have crucial roles in tissue repair and regeneration. In this study, we comprehensively investigated the proliferation and differentiation potential of infrapatellar fat pad stem cells (IFPSCs), synovium-derived stem cells (SDSCs), and bone marrow stem cells (BMSCs) from unpretreated knee joints in young rabbits, and after decellularized extracellular matrix (dECM) deposition by stem cell pretreatment *in vitro*.

**Methods:**

We also examined adhesion and differentiation effects of poly-L-lactic acid (PLLA) and poly-D, L-lactic acid (PDLLA) scaffolds after inoculation with the three stem cell types. We conducted osteogenic, adipogenic, and chondrogenic induction studies using three unpretreated stem cell groups, nine stem cell groups cross-preconditioned with different dECM types, and six stem cell groups cultured on nanofiber PLLA and PDLLA scaffolds. Staining and PCR analyses were then performed.

**Results:**

*In vitro* studies indicated that without pretreatment, IFPSCs exhibited the highest proliferation capacity, followed by SDSCs, while BMSCs had the lowest proliferation rate. After cross-pretreatment with dECMs from different sources, IFPSCs pretreated with IECM (decellularized extracellular matrix deposited by IFPSCs) showed the greatest proliferation. BMSCs displayed the highest osteogenic potential, while SDSCs and IFPSCs showed greater chondrogenic potential. No significant differences were observed in adipogenic potential among the three groups. BMSCs exhibited reduced osteogenic potential after pretreatment with all three dECMs, whereas IFPSCs and SDSCs showed enhanced osteogenic potential following SECM and IECM pretreatment, respectively. Additionally, all 3 cell types showed reduced lipogenic potential after pretreatment with the three dECM types. For chondrogenesis, BECM pretreatment were suitable for enhancing the chondrogenic potential of all 3 cell types. Furthermore, BMSCs and IFPSCs exhibited better adhesion and survival than SDSCs on electrospun scaffolds, which mimicked dECM structures. Besides, BMSCs and IFPSCs are more suitable for PLLA to promote osteogenic, adipogenic, and chondrogenic differentiation, whereas SDSCs are better suited for PDLLA.

**Discussion:**

Overall, it is anticipated that IFPSCs can be expanded with BECM pretreatment *in vitro*, and when combined with degradable nanofiber PLLA scaffolds *in vivo,* will facilitate better OA repair.

## 1 Introduction

Osteoarthritis (OA) is a widespread degenerative disease, characterized primarily by cartilage degradation (Martel-Pelletier et al., 2016). The absence of vascular, neural, and lymphatic networks in knee cartilage limits its capacity for complete regeneration and repair following injury (Guo et al., 2022). Current interventions for OA primarily consist of pharmacological treatments to alleviate pain and inflammation and surgical knee replacement in advanced stages (Uivaraseanu et al., 2022). However, these strategies do not prevent disease progression or promote cartilage regeneration. Hence, there is an urgent need to identify an effective strategy for optimal cartilage regeneration.

Mesenchymal stem cells (MSCs) are the most promising cell source for OA treatment due to their self-renewal and multi-differentiation potential (Song et al., 2020). Stem cell-based OA therapies require large numbers of high-quality stem cells, but *in vitro* expansion increases replicative senescence and reduces chondrogenic potential (Li and Pei, 2012), while *in vivo* transplantation leads to significant cell apoptosis (Eggenhofer et al., 2012), with few cells differentiating into chondrocytes. Given the varying chondrogenic potential of stem cells from different tissues (Pizzute et al., 2015), identifying an optimal stem cell source near the knee joint is crucial for effective cartilage repair. Furthermore, it is crucial to improve the chondrogenic potential *in vitro* and to identify a suitable biological scaffold for the support of seed cells, facilitating their use *in vivo*.

An increasing number of studies have highlighted the potential of bone marrow MSCs (BMSCs), synovium-derived stem cells (SDSCs), and infrapatellar fat pad stem cells (IFPSCs) as promising sources of cells for cartilage repair (Pizzute et al., 2015; Ma et al., 2018; Pei et al., 2022; Sun et al., 2018). Furthermore, numerous studies have demonstrated that the chondrogenic potential of SDSCs surpasses that of BMSCs (Pizzute et al., 2015; Ma et al., 2018). However, the relative chondrogenic potential of IFPSCs, in comparison to SDSCs and BMSCs, remains a subject of ongoing debate.

The application of decellularized extracellular matrix (dECM) deposited by stem cells in tissue engineering enables the *in vitro* acquisition of a large number of high-quality stem cells (Li et al., 2020). For example, Pei and colleagues (Li et al., 2020; Pei et al., 2023) demonstrated that adult SDSCs cultured on dECM deposited by adult SDSCs exhibited faster proliferation rates and enhanced chondrogenic potential compared to tissue culture plastic (Plastic) group. Furthermore, rabbit IFPSCs cultured on dECM deposited by adult SDSCs or urine-derived stem cells (UDSCs) demonstrated enhanced proliferation and, following *in vivo* implantation, exhibited superior cartilage repair compared to Plastic group (Pei et al., 2022). Thus, *in vitro* expansion of stem cells on dECM, coupled with biological scaffolds, appears to be a promising strategy for cartilage defect repair.

Electrical stimulation is crucial for cartilage (Liu et al., 2022) and bone (Zhang et al., 2024) regeneration; however, most biomaterials capable of generating electrical stimulation are either non-biocompatible or non-degradable (Azimi et al., 2020). A growing body of research emphasizes the piezoelectric properties, biocompatibility, and biodegradability of poly-L-lactic acid in tissue regeneration and repair (Capuana et al., 2022). Compared to non-piezoelectric PLLA combined with exercise therapy, piezoelectric PLLA with exercise therapy resulted in maximal hyaloid cartilage regeneration in rabbit cartilage defects (Liu et al., 2022). Our previous research showed that piezoelectric PLLA enhanced osteogenic differentiation of BMSCs compared to non-piezoelectric poly-D, L-lactide (PDLLA), particularly under mechanical stimulation (Zhang et al., 2024). However, it remains unclear whether the chondrogenic effect of all cell types on the piezoelectric material PLLA exceeds that of the non-piezoelectric material PDLLA.

Therefore, in this study, our aim was to identify the dECM-pretreated knee seed cells with the optimal chondrogenic potential, while also exhibiting best compatibility and chondrogenic capacity with electrospun nanofiber PLLA scaffolds to lay a solid theoretical foundation for tissue engineering applications for cartilage repair.

## 2 Materials and methods

### 2.1 IFPSC, SDSC, and BMSC isolation and expansion

This experiment was approved by the Ethics Committee of the General Hospital of Western Theater Command (ID: 2025EC3-ky004). All procedures during the operation were conducted in accordance with the requirements of the ethics committee.

Four New Zealand rabbits, aged 7–30 days, were obtained from Dacron Company (Chengdu, China). All animals in this experiment were raised in a clean and appropriate environment, with sufficient access to water and feed. Periauricular intravenous anesthesia with 1% sodium pentobarbital was administered for pain relief prior to sampling, and the rabbits were subsequently sacrificed through air embolization. IFPSCs were extracted from the infrapatellar fat pad, SDSCs from the synovial lining and ligament attachment in the knee joint, and BMSCs from the knee joint of the hind limb.

For IFPSC and SDSC extraction, tissues were cut into 0.5 mm × 0.5 mm sections and washed in phosphate buffered saline (PBS) to remove excess blood vessels and connective tissues. Tissues were then digested in a 2 mg/mL collagenase II solution (Biofrox, China) and incubated for 2 h at 37°C with agitation. Debris was filtered through a 70 μm cell filter (Biosharp, China), the filtrate centrifuged at 1,000 rpm for 10 min, cells resuspended in high-glucose Dulbecco’s Modified Eagle Medium (DMEM) (Gibco, China) plus 10% fetal bovine serum (Gibco, China), and added to T25 culture flasks (Corning, China).

For BMSC extraction, 5 mL of high-glucose DMEM was drawn into a syringe. Both ends of the femur were excised, and the bone marrow cavity washed until it turned white. Extracted contents were centrifuged at 1,000 rpm for 10 min, the supernatant discarded, and cells resuspended in the same medium. Cells were seeded into T25 culture flasks.

All cells were incubated at 37°C in 5% CO_2_ (Thermo Scientific, Massachusetts, USA). Cells were passaged at a 1:3 ratio when they reached 80%–95% confluence. Culture medium was changed every 3 days, and cells up to passage 3 (P3) were used for laboratory experiments.

### 2.2 Evaluating proliferation and surface phenotypes

When IFPSCs, SDSCs, and BMSCs reached approximately 90% confluence at P3, they were briefly digested with 0.25% EDTA-trypsin for approximately 2 min. Cells were then counted and seeded at 3,000 cells/well in 96-well plates, with each group seeded in triplicate for proliferation measurements over 1, 3, 5, and 7 days. After this, 10 µL of CCK-8 detection reagent (Biosharp) was added to wells, and plates incubated at 37°C for 2 h. Subsequently, absorbance at 450 nm was measured using an enzyme labeling device, and growth curves for 1, 3, 5, and 7 days plotted.

For immunofluorescence, P3 cells were also seeded at 3,000 cells/cm^2^ in six-well plates and cultured for 3 days. Cells were washed in PBS, fixed in 4% paraformaldehyde (Biosharp) for 20 min, and washed three times in PBS. They were then permeabilized in 0.5% Triton X-100 (Biosharp) in PBS for 15 min, followed by three more PBS washes. After blocking in 5% bovine serum albumin (BSA) (Biofrox, China) for 1 h, cells were incubated overnight at 4°C with the following primary antibodies: anti-CD44 rabbit polyclonal antibody (1:200, Beyotime, China), anti-CD73 rabbit polyclonal antibody (1:200, Beyotime), anti-CD90 rabbit monoclonal antibody (1:200, Beyotime), and anti-CD146 rabbit monoclonal antibody (1:200, Beyotime). After three PBS washes for 5 min each, cells were incubated with a fluorescein isothiocyanate-labeled goat anti-rabbit IgG (H + L) secondary antibody (Beyotime) at room temperature for 1 h. After three more PBS washes for 5 min each, nuclei were stained with 4′,6-diamidino-2-phenylindole (DAPI) (Beyotime) for 10 min. Finally, cells were washed three times in PBS for 5 min each and imaged under a fluorescence microscope (Olympus BX53, Japan).

### 2.3 Hematoxylin and eosin (H.E) staining

P3 cells were seeded at a density of 3,000 cells/cm^2^ in six-well plates and cultured for 3 days. Cells were then fixed in 4% paraformaldehyde for 30 min, stained with hematoxylin (Beyotime) for nuclear visualization, permeated, and counterstained with eosin (Beyotime). After dehydration and clearing, stem cell cytoplasm and nuclei were observed under inverted phase contrast microscopy (Olympus, Tokyo, Japan).

### 2.4 dECM preparation

Before seeding P3 cells in T25 culture flasks, they were coated as previously described (J Li and Pei, 2018). Briefly, flask bottoms were coated with 0.2% gelatin (McClean, China) in PBS and incubated at 37°C for 1 h, followed by three 5 min washes in PBS. Next, 1% glutaraldehyde (McClean) was added and incubated at room temperature for 30 min, followed by three additional 5 min washes in PBS. The coating was then treated with 1 M ethanolamine (McClean) for 30 min at room temperature and washed three times in PBS for 5 min each.

P3 cells were seeded in T25 culture flasks at 6,000 cells/cm^2^ in specified growth medium until 90% confluence. Subsequently, 250 mM 1000× L-ascorbic acid (Biosharp) in PBS was added to enhance extracellular matrix (ECM) deposition. The medium was changed every 2 days for 5–6 days. Finally, a cell extraction solution (0.5% Triton X-100 + 20 mM NH_4_OH in PBS) was applied for approximately 5 min until intact cells were no longer visible under inverted phase contrast microscopy (Olympus, Tokyo, Japan).

### 2.5 IFPSC, SDSC, and BMSC expansion on plastic and dECM

Three dECM types prepared as described previously (J Li and Pei, 2018) were used as substrates. P3 IFPSCs, SDSCs, and BMSCs were seeded onto culture flasks or these matrices at 3,000 cells/cm^2^, and incubated until 80%–90% confluence. The culture medium was changed every 3 days.

### 2.6 Synthesis of electrospun nanofiber scaffolds

PLLA (molecular weight = 160 kDa) and PDLLA (molecular weight = 150 kDa) nanofiber scaffolds were prepared as follows. PLLA and PDLLA were dissolved in a mixture of dichloromethane (Amresco, California, USA) and N,N-dimethylformamide (Amresco, California, USA) at a ratio of 7:3, achieving a concentration of 8%–10%. The mixture was heated to 37°C for 3–4 h in a water bath until materials were completely dissolved. Next, using a 10 mL syringe connected to an injection pump (WZ-50C2, China) set to 18 kV (DWP503–1AC, China), electrospinning was conducted at 40 μL/min for approximately 4 h at room temperature (Zhang et al., 2024).

### 2.7 dECM and nanofiber scaffold characterization

Morphology and fiber diameter dimensions of dECMs and electrospun nanofiber scaffolds were observed by scanning electron microscopy (SEM, FEI Quanta 250, Oregon, USA). As described previously (Zhang et al., 2024), samples were prepared as 1 cm × 1 cm sections, mounted on conductive plates, coated with approximately 10 nm of gold using a sputter coater, and observed under SEM.

The crystalline phase structures of PLLA and PDLLA nanofiber scaffolds were characterized via X-ray diffraction (XRD, France) over a scanning range of 5°–80°.

The piezoelectric properties of PLLA and PDLLA were assessed using a custom-built piezoelectric detector. Briefly, 1 cm × 1 cm PLLA and PDLLA samples were clamped and stretched between the device’s positive and negative chucks under controlled frequency and force. The piezoelectric output was measured using a voltage pre-amplifier (Keithley 6,514 System Electrometer, Beijing) and a charge detector (KD5002, Jiangsu) (Zhang et al., 2024).

### 2.8 Cell adhesion assays

The three stem cell types were seeded onto nanofiber PLLA and PDLLA scaffolds at 3,000 cells/cm^2^. After 3 days, cell adhesion morphology on nanofiber materials was observed under fluorescence microscopy (Olympus, Tokyo, Japan). Cells were fixed in 4% paraformaldehyde for 20 min, washed once in PBS, permeabilized in 0.5% Triton X-100 for 15 min, and blocked with 5% BSA for 1 h. Next, cells were stained with phalloidin-Alexa Fluor 488 (100 nM, Uelandy, China) for 1 h, washed three times in PBS for 5 min each, and incubated with DAPI (Beyotime) for 10 min. Finally, cells were washed three times in PBS for 5 min each.

### 2.9 Cell viability staining

P3 IFPSCs, SDSCs, and BMSCs were seeded on PLLA and PDLLA scaffolds at 3,000 cells/cm^2^ and cultured for 1 day. Cells were then stained with calcein-AM (C2015M, Beyotime, China) and propidium iodide (PI, C2015M, Beyotime, China); viable cells were stained with calcein-AM and apoptotic cells stained with PI. Fluorescence microscopy was used to capture images and record data.

### 2.10 Osteogenic, adipogenic, and chondrogenic differentiation evaluation

IFPSCs, SDSCs, and BMSCs at P3 (unpretreated) and P4 (dECM-amplified) were seeded into six-well plates at 3,000 cells/cm^2^ in high-glucose DMEM and the medium changed every 2 days. At 70% confluence, unpretreated cells underwent osteogenic differentiation in osteogenic differentiation medium (Maison, CTCC-001-MSCYD, China) for 3, 7, or 14 days. In contrast, dECM-pretreated cells were differentiated for 14 days, and cells cultured on electrospun nanofiber scaffolds were differentiated for 7 days. Additionally, unpretreated cells were subjected to 7- and 14-day differentiation protocols using adipogenic differentiation medium (Maison, CTCC-003-MSCYD) and chondrogenic culture medium (Maison, CTCC-002-MSCYD), respectively. On the other hand, dECM-pretreated cells were differentiated for 14 days, and cells cultured on electrospun nanofiber scaffolds for 7 days. Studies have demonstrated that stem cells differentiated on electrospun nanofiber materials achieve favorable results within 7 days (Zhang et al., 2024), whereas cells cultured without these materials require a longer duration. Plastic were used as the control for all dECM pretreatments, while PDLLA served as the control for piezoelectric PLLA.

#### 2.10.1 Alkaline phosphatase (ALP) and alizarin red S (ARS) staining

Osteogenic ALP activity in stem cells was assessed after 3 and 7 days of culture. Cells were fixed in 4% paraformaldehyde for 20 min, washed in PBS, and stained using a pluripotent stem cell ALP staining kit (Beyotime), containing ALP substrate, BCIP solution (1:300), and NBT solution (1:150), according to manufacturer’s instructions.

Calcified nodule activity in osteogenic samples was detected by ARS staining (Beyotime). After fixation in 4% paraformaldehyde for 30 min and washing in PBS, ARS staining was performed for 30 min, followed by repeated washing in PBS at least 3 times.

To semi-quantitatively analyze ARS staining in samples, 800 µL of 10% acetic acid in ddH_2_O was added to six-well plates and incubated for approximately 30 min. Subsequently, the acetic acid solution was collected and centrifuged at 3,000 rpm for 30 s. Next, samples were heated to 85°C and cooled on ice for 5 min before centrifugation at 12,000 rpm for 15 min. A portion of the liquid was neutralized in 200 µL of 10% ammonia solution. Then, absorbance at 405 nm was measured on a microplate reader (Thermo Scientific, Massachusetts, USA) in 96-well plates containing 100 µL of solution.

#### 2.10.2 Oil red O (ORO) staining

Samples were fixed in 4% paraformaldehyde, washed in PBS, stained in ORO (Beyotime) for 30 min, and rinsed in PBS until any residual dye was removed from the PBS.

For this process, 1 mL of isopropyl alcohol was added to wells for 5 min. Absorbance at 510 nm was measured using 100 µL of solution from wells for comparisons.

#### 2.10.3 Alcian blue (AB) staining for glycosaminoglycan

Chondrogenic samples were fixed in 4% paraformaldehyde and washed in PBS. After 30 min of AB staining, samples were washed in PBS until no residual dye was visible.

### 2.11 Real-time quantitative polymerase chain reaction (RT-qPCR)

We evaluated the *in vitro* differentiation potential of the three stem cell types cultured in Plastic, dECM, and nanofiber scaffolds for gene expression associated with osteogenesis (liver/bone/kidney alkaline phosphatase *(ALPL),* osteopontin *(OPN),* bone gamma carboxyglutamic acid containing protein *(BGLAP)*), adipogenesis (peroxisome proliferator-activated receptor gamma *(PPARγ),* CCAAT/enhancer binding protein alpha *(CEBPα),* and adipocyte determination and differentiation factor 1 *(ADD1)* (Pittenger et al., 1999)) and chondrogenesis (sex-determining region Y box 9 *(SOX9),* type II collagen *(COL2A1),* and aggrecan *(ACAN)*). TRIzol (Takara, China) was added to cells for 10 min. Lysates were transferred to centrifuge tubes, mixed with 200 µL of chloroform (Sinopharm, China), incubated for 5 min at room temperature, and then centrifuged at 12,000 rpm at 4°C for 10 min. The upper aqueous phase was transferred to an equal volume of ice-cold isopropyl alcohol (Sinopharm) and the mixture incubated at −20°C for 20 min. RNA was pelleted by centrifugation at 12,000 rpm for 10 min at 4°C, washed in 1 mL of 75% ethanol, and then recentrifuged at 12,000 rpm for 5 min at 4°C. The RNA pellet was air-dried and resuspended in 30 µL of diethylpyrocarbonate water at room temperature. RNA concentrations were quantified and cDNAs were synthesized using a reverse transcription kit (Thermo Scientific, Massachusetts, USA). PCR was conducted in a 20 µL reaction volume using the following cycling conditions: 2 min at 95°C, followed by 40 cycles of 15 s at 95°C and 30 s at 58°C. Primers are listed (Table 1).

**TABLE 1:** Primers of genes involved in cell-specific differentiation.

Name	Forward primer	Reverse primer
Osteogenesis-related genes		
*ALPL* *OPN* *BGLAP*	GTTCAGTGCGGTTCCAGACAA TGGCTTTCAATGGACTTACTCG TGATGCAAGCCTGACCTCC	CTGCAAGGACATCGCTTATCA CAGCTTTACCACAAACACCCG CCATAGCCCACGGCCAAAA
Adipogenesis-related genes		
*PPARγ* *CEBPα* *ADD1*	GGGTTGACCCTGAGAACCTC ATCTAGCCTCTCTCCGTCCC TGGAGAGAACCTGGACGAGA	GCAAATGATCCTCCACCCGA AAGCAACCCCCAAACCAGAA GGTACTCTCTAGCACGTCCG
Chondrogenesis-related genes		
*SOX9* *COL2A1* *ACAN*	GGACTACAAGTACCAGCCGC TGCAGGAGGGGAAGAGGTAT CTGGACTCTGGCAGTCTCAC	GGGGAATGGACCTCGCTCAT GCGAGGTCAGTAGGGCAG ATCTCTGCCCCAGAGGTCAC
Housekeeping gene		
*GAPDH*	AGTATGATTCCACCCACGGC	GATGGCCTTCCCGTTGATGA

*ALPL*, liver/bone/kidney alkaline phosphatase; *OPN*, osteopontin; *BGLAP*, bone gamma carboxyglutamic acid containing protein; *PPARγ*, peroxisome proliferator-activated receptor gamma; *CEBPα*, CCAAT/enhancer binding protein alpha; *ADD1*, adipocyte determination and differentiation factor 1; *SOX9*, sex-determining region Y box 9; *COL2A1*, collagen type 2; ACAN, aggrecan; *GAPDH*, glyceraldehyde-3-phosphate dehydrogenase.

### 2.12 Statistical analysis

All data are presented as the mean ± standard deviations. Statistical analysis was performed in GraphPad Prism 8 (GraphPad Software, CA, USA) to assess differences between multiple datasets. Normal and variance data were evaluated using Shapiro–Wilks and Levene’s tests for homogeneity, respectively. For normally distributed data with equal variance, one-way analysis of variance (ANOVA) with Bonferroni *post hoc* analysis was conducted. Alternatively, when assumptions of variance homogeneity were violated, one-way ANOVA (Welch’s F tests) with Games–Howell *post hoc* analysis was used. Statistical significance was accepted at p < 0.05, with mean differences denoted by letters (a, b, and c). Within-group comparisons label the group with the lowest mean as “a” and sequentially compare others by mean differences until the highest-mean group is identified. In comparisons among groups of the same cell type groups: # denotes significant differences (P < 0.05) vs Plastic (PL); $ indicates significant differences (P < 0.05) between BECM and IECM, and between BECM and SECM; ^ represents significant differences (P < 0.05) between IECM and SECM groups.

## 3 Results

### 3.1 Isolation, culture, and phenotypic characterization of IFPSCs, SDSCs, and BMSCs

**FIGURE 1:**
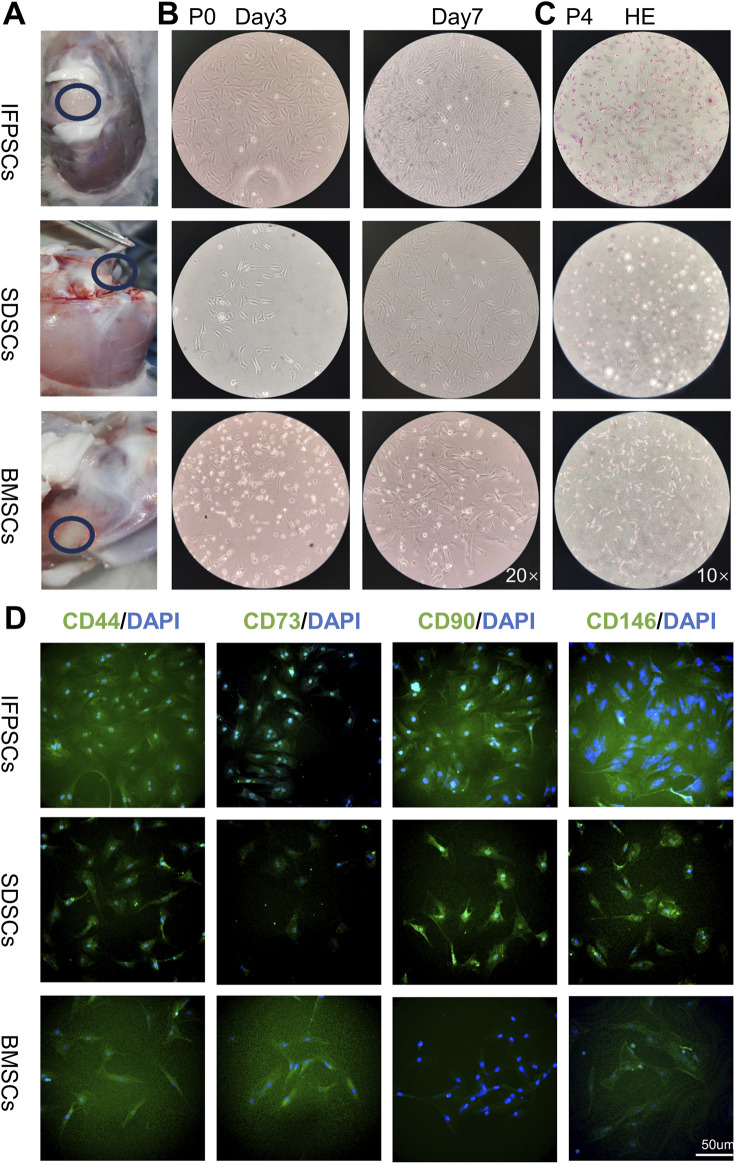
**(A)** Stem cell extraction sites from young rabbits. **(B)** P0 cell growth morphology on days 3 and 7 under a ×20 magnification inverted microscope. **(C)** H.E staining images show P4 infrapatellar fat pad stem cells (IFPSCs), synovium-derived stem cells (SDSCs), and bone marrow stem cells (BMSCs). **(D)** Immunofluorescence images show CD44, CD73, CD90, and CD146 expression on stem cell surfaces. Scale bar = 50 µm.

IFPSCs were isolated from infrapatellar fat pad tissues, SDSCs from the knee ligament and synovial lining tissues, and BMSCs from the bone marrow cavity of the knee joint (Figure 1A). All three P0 cell types exhibited colony swirl-like growth, resembling fibroblasts with long spindle shapes. By day 3, IFPSC growth rates were significantly higher than SDSC and BMSC rates, with IFPSCs reaching >80% confluence by day 7 (Figure 1B). HE staining revealed consistent cell morphology across all 3 cell types during passage, characterized by long spindle shapes and oval nuclei. The cytoplasm appeared red, while nuclei were blue-purple (Figure 1C). Immunofluorescence confirmed positive surface antigen marker expression (CD44, CD73, CD90, and CD146) in IFPSCs and SDSCs, consistent with expected stem cell surface antigen phenotypes [67]. However, while CD44, CD73, and CD146 showed positive expression in BMSCs, CD90 expression was weak or even absent (Figure 1D). The initial cell densities are identical.

### 3.2 dECM and nanofiber scaffold characterization

**FIGURE 2:**
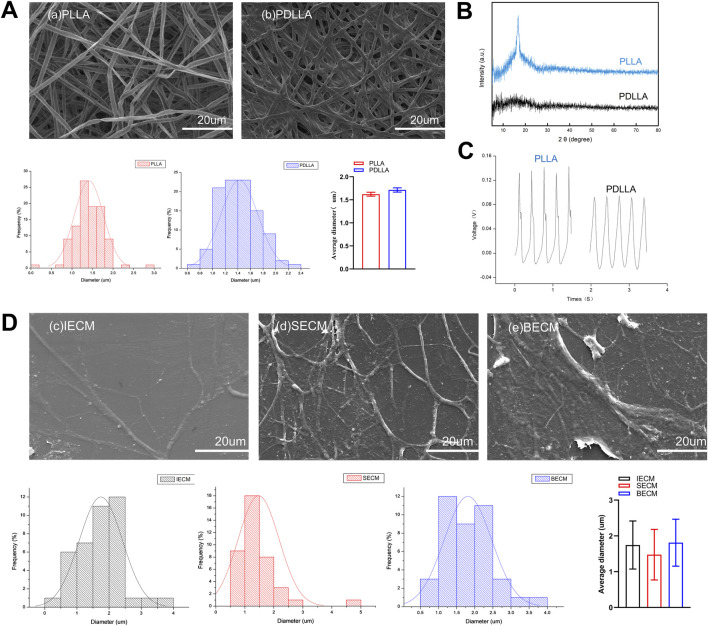
**(A)** SEM images show electrospun nanofiber poly-L-lactic acid (PLLA) and poly-D, L-lactic acid (PDLLA) scaffolds. **(B)** The characteristic peak of the piezoelectric nanomaterial PLLA was analyzed using X-ray diffraction (XRD). **(C)** Comparison of the piezoelectric voltage output between PLLA and PDLLA. **(D)** SEM images show decellularized extracellular matrices (dECMs) deposited by IFPSCs, SDSCs, and BMSCs.

Electrospun nanofiber PLLA and PDLLA scaffolds and dECM morphology were observed under SEM. Nanofiber scaffolds exhibited a 3D structure with nondirectional arrangements. PLLA and PDLLA nanofiber scaffold diameters were 1.41 ± 0.35 µm and 1.42 ± 0.29 µm, respectively. No significant diameter differences were detected between groups (Figure 2A). Additionally, XRD analysis identified a characteristic peak at 17.1° in the β-phase of piezoelectric PLLA, which was absent in PDLLA (Figure 2B). Piezoelectric testing revealed that both PLLA and PDLLA exhibited piezoelectricity, though the extremely low voltage in PDLLA may result from triboelectric effects (Figure 2C).

Concurrently, dECMs formed by the three stem cell types displayed similar 3D scaffold network structures. Similarly, IECM (dECM deposited by IFPSCs) with 3D nanofiber structures had a fiber diameter of 1.74 ± 0.67 µm, SECM (dECM deposited by SDSCs) had a diameter of 1.48 ± 0.70 µm, and BECM (dECM deposited by BMSCs) had a diameter of 1.81 ± 0.66 µm. No significant differences in fiber diameters were recorded across the three dECM types (Figure 2D). Interestingly, the electrospun nanofiber scaffold diameter was comparable to dECMs deposited by stem cells, but no significant difference was recorded.

### 3.3 Cell growth on dECM and electrospun nanofiber PLLA and PDLLA scaffolds

**FIGURE 3:**
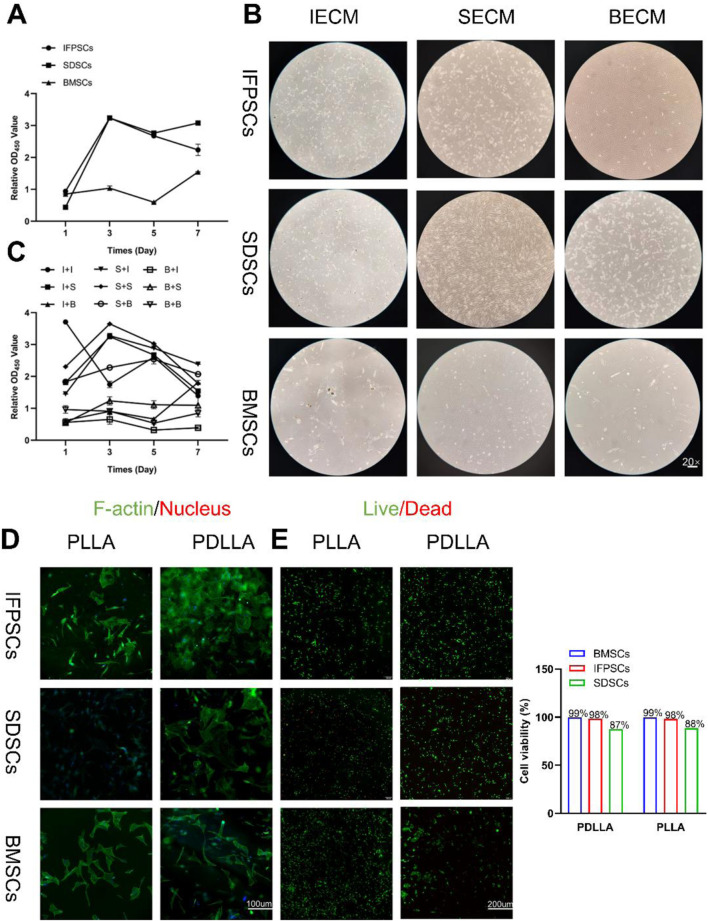
**(A)** Original stem cell proliferation rates were measured using a CCK-8 kit at 1, 3, 5, and 7 days. **(B)** Images of cells on dECM. **(C)** dECM pretreated-stem cell proliferation rates were measured using a CCK-8 kit at 1, 3, 5, and 7 days. (Key; I + I: IFPSCs + IECM (dECM deposited by IFPSCs); I + S: IFPSCs + SECM (dECM deposited by SDSCs); I + B: IFPSCs + BECM (dECM deposited by BMSCs); S + I: SDSCs + IECM; S + S: SDSCs + SECM; S + B: SDSCs + BECM; B + I: BMSCs + IECM; B + S: BMSCs + SECM; B + B: and BMSCs + BECM). **(D)** Cell adhesion morphology on PLLA and PDLLA nanofiber scaffolds. **(E)** Cell survival on electrospun nanofiber scaffolds made from PLLA and PDLLA. The cell viability was quantified using Image J software.

P4 IFPSC, SDSC, and BMSC proliferation rates were measured in Plastic after 1, 3, 5, and 7 days. IFPSCs exhibited the fastest proliferation rates during the first 3 days, followed by SDSCs, with BMSCs showing the slowest rates (Figure 3A). This observation was consistent with microscopy images taken of early primary cell cultures on day 3 (Figure 1B). IFPSCs and SDSCs entered rapid logarithmic growth phases within 3–4 days, whereas BMSCs reached this phase after 7 days (Figure 3A).

Cells cultured on dECM exhibited an elongated, spindle-shaped morphology, aligning along dECM fibers (Figure 3B). Subsequently, proliferation rates of the three stem cell lines treated with three different dECMs were measured. Following dECM pretreatment, cell proliferation rates improved to some extent. Interestingly, BMSCs, which initially exhibited the slowest proliferation rates without treatment, maintained relatively sluggish proliferation rates even after dECM treatment when compared with the other groups. Moreover, IFPSCs and SDSCs cultured on BECM showed lower proliferation rates than cells cultured on IECM or SECM (Figure 3C).

The three stem cell types were cultured on previously prepared nanofiber scaffolds, with cell adhesion observed under fluorescence microscopy on day 3. Green fluorescence indicated F-actin (phalloidin-stained) and blue indicated nuclei (DAPI-stained). Unlike monolayer growth in Plastic, all stem cells adhered to and overlapped with materials. Most cells adhered to nanofiber scaffolds and exhibited elongated spindles or diamond shapes (Figure 3D). All stem cell types demonstrated good biocompatibility with nanofiber scaffolds. Notably, BMSCs and IFPSCs showed higher survival rates (99% and 98% respectively) than SDSCs (88%), suggesting their superior suitability for PLLA and PDLLA (Figure 3E). Besides, no significant difference in survival rates was observed between PLLA and PDLLA across all stem cell types, indicating their equivalent effect on cell survival (Figure 3E).

### 3.4 Assessing osteogenic differentiation

**FIGURE 4:**
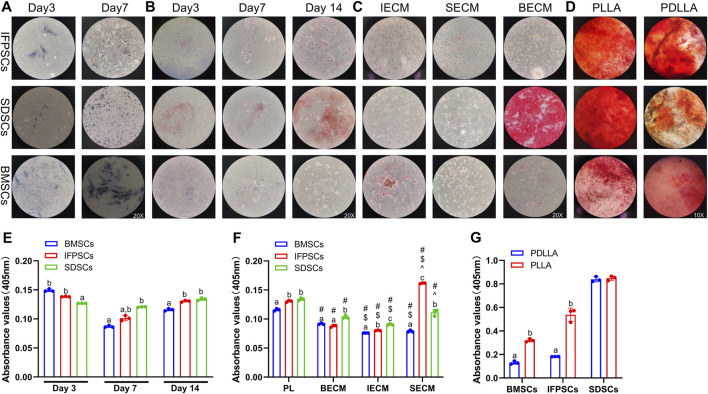
**(A)** ALP staining shows cells on days 3 and 7. **(B)** ARS staining shows cells grown in tissue culture plastic (Plastic) on days 3, 7, and 14. **(C)** ARS staining shows cells grown on dECM on day 14 of osteogenesis. **(D)** ARS staining data show osteogenic induction in cells grown on electrospun nanofiber scaffolds on day 7. **(E)** Semi-quantitative ARS staining analysis of osteogenic induction in cells grown in Plastic. **(F)** Semi-quantitative ARS staining analysis of osteogenic induction in cells grown on dECM. **(G)** Semi-quantitative ARS staining analysis of osteogenic induction in cells grown on nanofibrous PLLA and PDLLA scaffolds.

**FIGURE 5:**
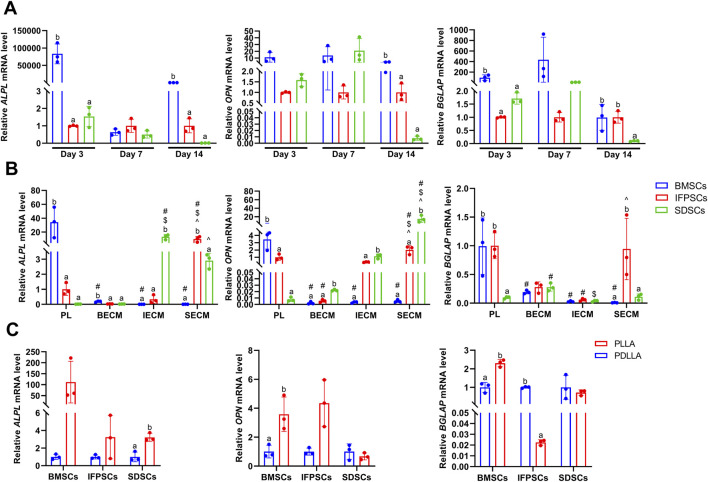
**(A)** Gene expression associated with osteogenesis at 3, 7, and 14 days in cells grown in Plastic. **(B)** Gene expression at 14 days of osteogenesis in IFPSCs, SDSCs, and BMSCs pretreated with dECMs, compared to Plastic (PL). **(C)** Gene expression in IFPSCs, SDSCs, and BMSCs on nanofiber PLLA scaffolds after 7 days of osteogenesis, compared to PDLLA.

ALP staining was performed at days 3 and 7 after the osteogenic induction of unpretreated stem cells. At day 3, BMSCs exhibited extensive purple staining, followed by IFPSCs, and SDSCs which showed the least intense staining. However, by day 7, there were no significant differences in staining intensity between cell types. ALP staining at day 7 was more pronounced than at day 3 of osteogenic differentiation (Figure 4A). On day 3, BMSCs exhibited more red staining than the other cell types, but this difference was not significant on day 7. By day 14, SDSCs showed more calcified nodules than BMSCs and IFPSCs (Figure 4B). As shown in the semi-quantitative ARS staining staining (Figure 4E), BMSCs and IFPSCs exhibited more calcification than SDSCs on day 3 after osteogenic induction. Interestingly, SDSCs formed the most calcified nodules by day 7. By day 14, IFPSCs and SDSCs displayed calcification comparable to or exceeding that of BMSCs. Furthermore, BMSCs exhibited higher osteogenic mRNA levels of *ALPL*, *OPN*, and *BGLAP* compared to IFPSCs and SDSCs on day 3 and 14, while no significant differences were observed among the 3 cell types on day 7 (Figure 5A).

The *in vitro* osteogenic differentiation results suggest that BMSCs from young rabbit knee joints exhibited stronger osteogenic potential than IFPSCs and SDSCs, with no significant difference observed between IFPSCs and SDSCs. The ALP and ARS staining on day 3 aligned with osteogenic gene expression. However, discrepancies observed on day 7 and 14 may reflect dynamic gene regulation during osteogenesis and the involvement of multiple genes in calcification.

After 14 days of osteogenic induction in cells pretreated with dECM, no significant differences in ARS staining were detected among the three types of dECM-treated IFPSCs. However, in SDSCs, cells cultured on BECM showed more intense staining than in the other groups. By day 14, BMSCs pretreated with IECM exhibited the most intense ARS staining. After SECM pretreatment, no significant differences in staining intensity were recorded among groups; however, among cells pretreated with BECM, those pretreated with SDSC exhibited the most staining intensity (Figure 4C). Among IFPSCs pretreated with dECM for 14 days, the IFPSC + SECM group exhibited the strongest osteogenic ability. After 14 days of dECM pretreatment, both SDSC + BECM and SDSC + SECM groups exhibited stronger osteogenic ability when compared with the SDSC + IECM group. Among dECM-pretreated BMSCs, the BECM-pretreated group exhibited stronger osteogenic ability after 14 days of osteogenesis when compared with ECM and SECM groups. Among the 3 cell types, SDSCs exhibited the strongest osteogenic ability after IECM pretreatment. IFPSCs demonstrated the strongest osteogenic ability following SECM pretreatment. SDSCs and BMSCs showed comparable osteogenic ability after BECM pretreatment (Figure 4F). Compared to Plastic group, *ALPL*, *OPN*, and *BGLAP* expression was downregulated in BMSCs following all dECMs pretreatments during osteogenesis. In contrast, SECM-pretreated IFPSCs exhibited upregulated *ALPL* and *OPN*, suggesting enhanced osteogenic potential, while BECM and IECM pretreatment had no significant effect on their osteogenic potential. For SDSCs, *ALPL* and *OPN* were upregulated after IECM and SECM pretreatment, whereas *BGLAP* expression remained unchanged compared to the Plastic group (Figure 5B). Notably, *ALPL* and *BGLAP* expression were upregulated in the IFPSC + SECM group compared to the SDSC + SECM group, suggesting greater osteogenic potential despite the downregulation of *OPN* expression (Figure 5B).

After 7 days of osteogenic induction on PLLA, SDSCs and IFPSCs exhibited stronger staining than BMSCs. In the PDLLA group, IFPSCs showed the most intense red staining (Figure 4D). Furthermore, on PLLA, SDSCs exhibited the strongest osteogenic ability, followed by IFPSCs, while BMSCs showed the weakest ability (Figure 4G). Similarly, on PDLLA, SDSCs demonstrated the strongest osteogenic potential, with IFPSCs and BMSCs exhibiting stronger osteogenic ability on PLLA than on PDLLA (Figure 4G). The ARS staining results aligned with the expression trends of osteogenic genes. *ALPL*, *OPN*, and *BGLAP* expression levels were significantly higher in BMSCs and IFPSCs combined with PLLA compared to PDLLA. In contrast, SDSCs showed no significant differences in *OPN* and *BGLAP* expression between PLLA and PDLLA, despite a decrease in *ALPL* expression (Figure 5C). The results indicate that IFPSCs and BMSCs combined with piezoelectric PLLA exhibited superior osteogenic effects compared to PDLLA, whereas SDSCs showed no significant difference in osteogenic potential between the two materials.

### 3.5 Assessing adipogenic differentiation

**FIGURE 6:**
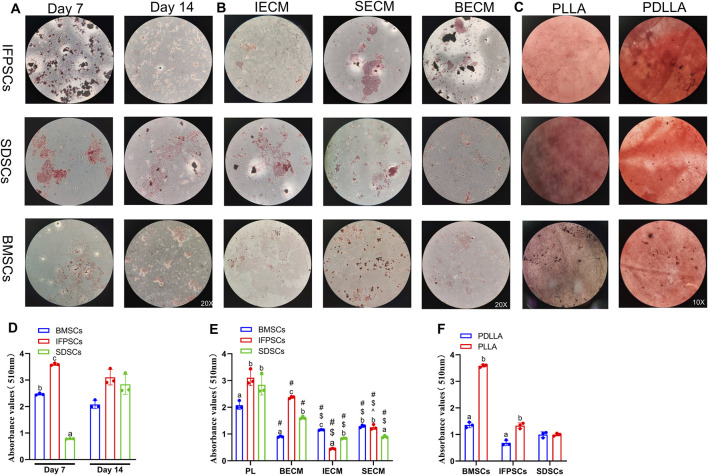
**(A)** Cells grown in Plastic for 7 or 14 days stained with ORO. **(B)** Cells grown on dECM stained with ORO on day 14 of adipogenesis. **(C)** Adipogenic induction in cells grown on electrospun nanofiber scaffolds stained with ORO on day 7. **(D)** Semi-quantitative ORO staining analysis of adipogenic induction in cells grown in Plastic (PL) group. **(E)** Semi-quantitative ORO staining analysis of adipogenic induction in growing cells on dECM. **(F)** Semi-quantitative ORO staining analysis of adipogenic differentiation in growing cells on nanofibrous PLLA scaffold, compared to PDLLA.

**FIGURE 7:**
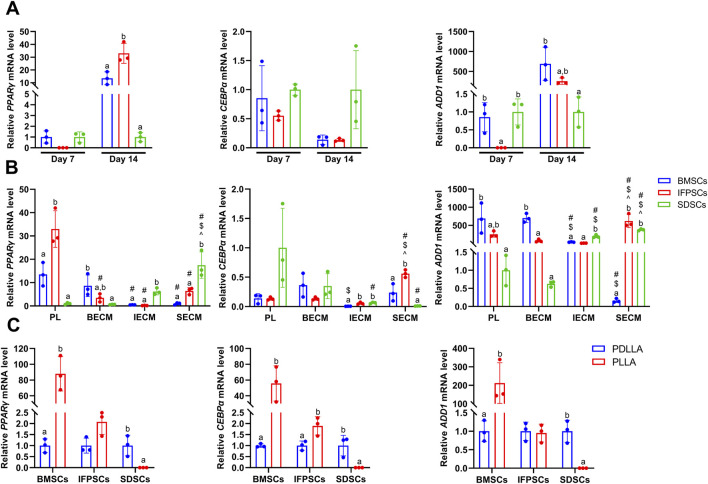
**(A)** Gene expression associated with adipogenesis at day 7 and 14 in cells grown in Plastic (PL). **(B)** Gene expression at day 14 of adipogenesis in IFPSCs, SDSCs, and BMSCs pretreated with dECM, compared to PL group. **(C)** Gene expression in IFPSCs, SDSCs, and BMSCs on nanofiber PLLA scaffold after 7 days of adipogenesis, compared to PDLLA.

Adipogenic induction in unpretreated cells in Plastic was observed at 7 and 14 days, with lipid droplets visible in all 3 cell types (Figure 6A). By day 7, IFPSCs exhibited more lipid droplets than the other cell types. However, by day 14, no significant differences in ORO staining was observed among cell types (Figure 6A). Further semi-quantitative ORO staining analysis revealed adipogenic ability in the 3 cell types without pretreatment. IFPSCs exhibited the strongest lipid-formation, followed by BMSCs, with SDSCs showing the weakest formation at day 7 (Figure 6D). Interestingly, the expression of *PPARγ* and *CEBPα* showed no significant differences among the 3 cell types on day 7 of adipogenic induction (Figure 7A). Although no significant differences in ORO staining (Figure 6D) and *CEBPα* expression (Figure 7A) were recorded at day 14, the adipogenic-related genes *PPARγ* and *ADD1* were more highly expressed in IFPSCs and BMSCs than in SDSCs (Figure 7A). These findings suggest dynamic gene regulation during adipogenesis, with BMSCs, IFPSCs, and SDSCs exhibiting comparable adipogenic potential.

After 14 days of adipogenic induction with dECM pretreatment, the IFPSC + SECM and IFPSC + BECM groups exhibited increased lipid droplet formation compared to the IFPSC + IECM group (Figure 6B). Similarly, the SDSC + IECM group demonstrated superior adipogenic potential than the other dECM groups, while the BMSC + SECM group exhibited increased lipid droplet formation compared to the others (Figure 6B). Consistent with microscopic observations, semi-quantitative ORO staining showed that dECM-pretreated IFPSCs exhibited the highest lipid formation in the IFPSC + BECM group, followed by the IFPSC + SECM group, with the lowest in the IFPSC + IECM group (Figure 6E). Compared with the other two dECM groups, the SDSC + BECM group exhibited greater lipid formation (Figure 6E). Besides, lipid droplet formation in the BMSC group following SECM or IECM pretreatment was lower than in the BMSC + BECM group (Figure 6E). Consistent with the ORO staining results, the expression of *PPARγ*, *CEBPα*, and *ADD1* was upregulated in the IFPSC + SECM group compared to other dECMs pretreatment (Figure 7B). Surprisingly, the expression of *PPARγ* and *ADD1* was upregulated in the SDSC + SECM and SDSC + IECM groups compared to the SDSC + BECM group (Figure 7B). In addition, the expression of *PPARγ* and *ADD1* in the BMSC + IECM and BMSC + SECM groups appeared slightly decreased compared to the BMSC + BECM group (Figure 7B). Interestingly, lipid droplet formation in dECM-pretreated IFPSCs, SDSCs and BMSCs was lower than in the Plastic group (Figure 6E). Consistent with semi-quantitative ORO staining, *PPARγ* expression in IFPSCs was significantly downregulated after all dECM pretreatments, despite inconsistent trends in *CEBPα* and *ADD1* expression (Figure 7B). Compared to the Plastic group, *PPARγ* and *ADD1* expression in SDSCs was upregulated following IECM and SECM pretreatment (Figure 7B). Additionally, the expression of *PPARγ* and *ADD1* in BMSCs was significantly downregulated following all IECM and SECM pretreatments (Figure 7B). However, no significant change in lipogenic potential of SDSCs and BMSCs was observed after BECM pretreatment (Figure 7B). In brief, pretreatment of the same cells with different dECMs, as well as pretreatment of different cells with the same dECMs, resulted in changes to their adipogenic potential. dECM pretreatment in SDSCs, IFPSCs, and BMSCs appeared to downregulate their lipid formation capacity.

The 3 cell types were differentiated into adipocytes on PLLA and PDLLA scaffolds for 7 days, with ORO staining slightly stronger on PDLLA than PLLA scaffolds under microscopy (Figure 6C). Semi-quantitative ORO staining showed that IFPSCs and BMSCs exhibited stronger effects on lipid formation on PLLA than PDLLA, while SDSCs showed comparable adipogenic effects on both (Figure 6F). Consistent with the semi-quantitative results, BMSCs on PLLA exhibited higher expression of lipogenic genes *PPARγ*, *CEBPα*, and *ADD1* than those on PDLLA. Similarly, IFPSCs on PLLA showed an increasing trend in *PPARγ* and *CEBPα* expression compared to PDLLA. Interestingly, SDSCs on PLLA had significantly lower expression of *PPARγ*, *CEBPα*, and *ADD1* than those on PDLLA (Figure 7C). In summary, PLLA appears to enhance the adipogenic potential of BMSCs and IFPSCs compared to non-piezoelectric PDLLA, while PDLLA is more favorable for SDSCs.

### 3.6 Assessing chondrogenic differentiation

**FIGURE 8:**
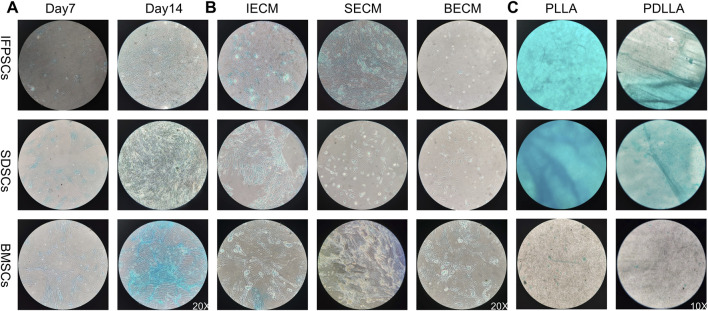
**(A)** Cells grown in Plastic (PL) on days 7 and 14 were stained with AB staining. **(B)** Cells grown on dECM were stained with AB staining on day 14 of chondrogenic induction. **(C)** Chondrogenic induction in cells grown on PLLA nanofiber scaffold stained with AB staining on day 7.

**FIGURE 9:**
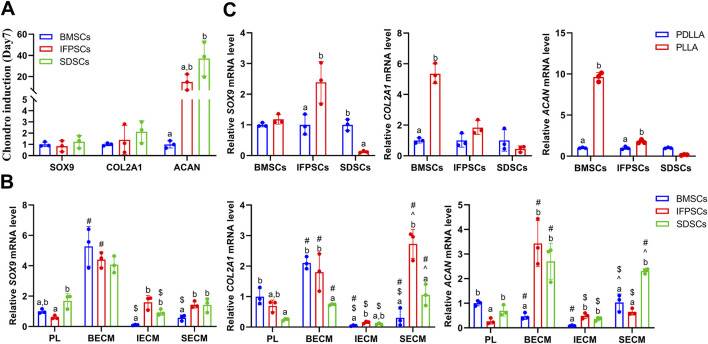
**(A)** Gene expression associated with chondrogenesis at days 7 in cells grown in Plastic (PL). **(B)** Gene expression at day 14 of chondrogenesis in IFPSCs, SDSCs, and BMSCs pretreated with dECM, compared to PL group. **(C)** Gene expression in IFPSCs, SDSCs, and BMSCs on nanofiber PLLA scaffold after 7 days of chondrogenesis, compared to PDLLA.

No significant differences in AB staining among all types of unpretreated stem cells after 7 days of chondrogenic differentiation. By day 14, SDSCs and BMSCs exhibited deeper blue staining than IFPSCs, indicating more intense glycosaminoglycan production. Ab staining intensity increased from day 7–14 across all cell types (Figure 8A). After 7 days of chondrogenic induction, the expression levels of *SOX9*, *COL2A1*, and *ACAN* were higher in IFPSCs and SDSCs compared to BMSCs (Figure 9A). Moreover, at 14 days of induction, *SOX9* and *ACAN* exhibited the highest expression in SDSCs. Notably, there was no significant difference in chondrogenic potential between BMSCs and IFPSCs (Figure 9B). In conclusion, SDSCs appears to exhibit the highest chondrogenic potential, followed by IFPSCs, while BMSCs show the least chondrogenic capacity.

In IFPSCs pretreated with dECM and chondrogenically differentiated for 14 days, the IFPSC + SECM group exhibited the darkest staining, whereas the IFPSC + BECM group showed the lightest. Similarly, after 14 days of chondrogenesis, SDSCs pretreated with dECM exhibited the most intense staining in the SDSC + IECM group, while the SDSC + BECM group exhibited the least intense staining. BMSCs pretreated with dECM for 14 days showed the deepest staining in the BMSC + SECM group, with no significant differences between the other two groups under microscope (Figure 8B). Interestingly, the expression levels of *SOX9*, *COL2A1*, and *ACAN* were significantly upregulated in all cell types during chondrogenic induction following BECM pretreatment compared to the Plastic group (Figure 9B). It is worth noting that, in contrast to the upregulated expression of chondrogenic genes in IFPSCs and SDSCs pretreated with SECM, the expression of *SOX9*, *COL2A1*, and *ACAN* in BMSCs was downregulated following pretreatment with both IECM and SECM (Figure 9B). These results indicate that all three types of stem cells are suitable for pretreatment with BECM to enhance their chondrogenic potential.

After 7 days of chondrogenic induction on PLLA and PDLLA scaffolds, IFPSCs and SDSCs exhibited stronger Ab staining than BMSCs on both. Notably, while no significant differences in BMSC staining intensity were identified between PLLA and PDLLA, IFPSCs and SDSCs showed slightly stronger staining on PLLA than on PDLLA under microscope (Figure 8C). It is interesting to note that BMSCs and IFPSCs on PLLA exhibited higher expression of *SOX9*, *COL2A1*, and *ACAN* compared to those on PDLLA. However, SDSCs demonstrated a lower ability to differentiate into chondrocytes on PLLA than on PDLLA (Figure 9C). These findings indicate that BMSCs and IFPSCs are more compatible with PLLA for cartilage regeneration, while SDSCs show better suitability for PDLLA, likely due to differential cellular responses to the materials.

## 4 Discussion

The selection of appropriate seed cells for cartilage regeneration and repair provides considerable advantages, underscoring the importance of understanding the biological characteristics of various cell types. In this study, using cells derived from the knee joints of young rabbits, we investigated differences in IFPSC, SDSC, and BMSC proliferation rates and osteogenic, adipogenic, and chondrogenic potential. All stem cell types proliferated rapidly without pretreatment, with IFPSCs showing the highest proliferation rates, and BMSCs the lowest. These rates significantly improved after pretreatment with respective dECM types. Interestingly, the proliferation rate of all cells cultured on BECM was significantly lower than that on IECM and SECM. This phenomenon may be linked to embryonic origins shared by IFPSCs and SDSCs (Bobacz et al., 2008). However, differences in cell phenotypes were observed among the three stem cell types. CD90 is typically, positively expressed in human MSCs (Li et al., 2020), but we observed weak or absent CD90 expression in BMSCs from young rabbits. These results align with a previous study reporting weak CD90 expression in rabbit BMSCs (Lapi et al., 2008). Such variability in CD90 expression (Peister et al., 2004) may be attributed to factors such as species, age, extraction method, *in vitro* amplification, and donor culture conditions.

Additionally, we observed that BMSCs exhibited lower chondrogenic capacity than IFPSCs and SDSCs. Among the three dECMs, BECM appeared to most effectively enhance the chondrogenic potential of BMSCs, IFPSCs, and SDSCs compared to the Plastic group. This result is similar to Li et al.'s finding (Li et al., 2020) that adult SDSCs exhibited significantly higher chondrogenic potential after expansion on dECM deposited by UDSCs, which lack chondrogenic ability, or dECM deposited by adipose-derived stem cells with limited chondrogenic capacity, compared to SECM. This indicates that dECM deposited by stem cells with limited chondrogenic potential may enhance the chondrogenic capacity of expanded cells, compared to the dECM deposited by stem cells with higher chondrogenic capacity.

Beyond differences in chondrogenic potential, the osteogenic potential among the 3 cell types was also markedly distinct. Specifically, BMSCs exhibited greater osteogenic potential than IFPSCs and SDSCs. Notably, while *ALPL* is an early marker of osteogenesis, it was consistently expressed from day 3–14 in our study, aligning with the findings of Li et al. (2024). The semi-quantitative osteogenesis results do not align with osteogenic gene expression, likely due to ongoing cell apoptosis during osteogenic differentiation (Song et al., 2009). Interestingly, BMSCs in young rabbits exhibit a downregulation of osteogenic potential following preconditioning with all three dECMs. This finding contrasts with the upregulation of *ALPL* during osteogenesis of Adult BMSCs on adult BECM, as reported by Pei et al. (2011), likely due to differences in donor cell origin and species. Besides, IFPSCs and SDSCs from young rabbits exhibited upregulation of osteogenic potential on SECM, consistent with the upregulation of *ALPL* observed in the osteogenic process of fetal SDSCs after preconditioning on fetal SECM, as reported by Pei et al. (2024). The osteogenic potential of IFPSCs expanded on IECM and BECM was comparable to that of the plastic group, consistent with the mineralization of fetal SDSCs on different dECMs (Li et al., 2015).

Previous studies have shown that the adipogenic differentiation ability of adult BMSCs pretreated with adult BECM (Pei et al., 2011), adult IFPSCs with adult IECM (Wang et al., 2021), and adult SDSCs with adult SECM (Pei et al., 2023) decreased to varying degrees. Similarly, we observed a varying degree of downregulation in the lipid-forming ability of the three stem cells following dECM pretreatment. Fetal cells appear to respond differently to dECM than adult cells, with reports indicating that fetal SDSCs pretreated with SECM enhanced lipid droplet formation (Li et al., 2015). In summary, osteogenic and adipogenic differentiation potential varies with both the type of dECM and the cell source, warranting further investigation into the underlying molecular mechanisms.

To optimize the combination of dECM pretreatment and piezoelectric PLLA for enhanced cartilage regeneration, it is essential to identify the stem cell type in the knee joint that exhibits the best biocompatibility and chondrogenic potential when combined with PLLA. This seed cell should also demonstrate optimal chondrogenic potential with the most suitable dECM type. Ultimately, the best seed cell-dECM combination will be selected for future cartilage tissue repair applications. Our results found that BMSCs and IFPSCs derived from the knee joint of young rabbits exhibit superior osteogenic, adipogenic, and chondrogenic differentiation *in vitro* on PLLA compared to PDLLA, whereas SDSCs show greater differentiation on PDLLA. Notably, the expression levels of *ALPL*, *OPN*, and *BGLAP* during osteogenic differentiation of BMSCs on PLLA were higher than those on PDLLA, consistent with our team’s previous findings (Zhang et al., 2024). Furthermore, Liu et al. (2022) reported no significant differences in the expression of chondrogenic genes *SOX9*, *COL2A1*, and *ACAN* in ADSCs cultured on piezoelectric versus non-piezoelectric PLLA under non-pressure stimulation, a result similar to our findings with SDSCs on biomaterials. Interestingly, SDSCs exhibit a distinct response to the same biomaterials compared to BMSCs and IFPSCs, likely due to differential cellular responses to the same microenvironment (Pei et al., 2023). Moreover, we observed that all stem cell types adhered to and differentiated effectively on nanofiber scaffolds, with SDSCs exhibiting slightly lower survival than BMSCs and IFPSCs.

For clinical applications, IFPSC extraction causes significantly less patient discomfort than BMSC extraction, which carries donor site morbidity, wound infection, and sepsis risks (Pittenger et al., 1999). In contrast, IFPSCs not only proliferate at greater rates than SDSCs but also maintain their differentiation potential over prolonged passages (Khan et al., 2009). Overall, IFPSCs demonstrate strong potential for cartilage repair applications.

However, this study has certain limitations. Further research is needed to assess the multi-differentiation potential of the three MSC types in knee joints across varying ages and health conditions. Additionally, due to substantial cell requirements from rabbit knee joints for this study, multiple rabbits were used. Therefore, the three types of knee stem cells used in subsequent experiments were obtained from donor-matched rabbits, ensuring greater comparability in their biological characteristics. While these stem cells demonstrated good viability and differentiation on nanofibrous materials *in vitro*, further research is required to assess whether *in vivo* and *in vitro* augmentation of cells pretreated with dECM on piezoelectric materials exhibits synergistic chondrogenic potential. Furthermore, it remains to be determined whether the molecular mechanism underlying chondrogenesis in stem cells, characterized by increased TGF-β receptor two expression following dECM pretreatment (Pei et al., 2011), can be further promoted by piezo-enhanced calcium influx and the resulting increase in TGF-β1 levels (Liu et al., 2022).

## 5 Conclusion

Taken together, among the three stem cell types isolated from young rabbit knee joints, BMSCs exhibited the strongest osteogenic potential, while SDSCs and IFPSCs demonstrated superior chondrogenic potential, with no significant differences in adipogenic potential. BMSCs showed reduced osteogenic potential after all dECM pretreatments, whereas IFPSCs and SDSCs exhibited enhanced osteogenesis following SECM and IECM pretreatment, respectively. All cell types displayed reduced lipogenic potential after dECM pretreatment. Notably, BECM pretreatment enhanced chondrogenic potential across all stem cell types, particularly in IFPSCs, which demonstrated strong biocompatibility and chondrogenic potential on PLLA. Future strategies may involve BECM-pretreated IFPSCs integrated into biocompatible, degradable PLLA scaffolds *in vivo*, offering a promising approach for cartilage tissue engineering and regeneration.

## Data Availability

The datasets presented in this study can be found in online repositories. The names of the repository/repositories and accession number(s) can be found in the article/supplementary material.
