# Correction: A comparative analysis of stem cells derived from young rabbit knee joints: the potentially superior performance of decellularized extracellular matrix pretreated infrapatellar fat pad stem cells on nanofiber scaffolds

**DOI:** 10.3389/fcell.2026.1779159

**Published:** 2026-02-06

**Authors:** Zhixin Wei, Qingqing Yu, Qingyun Xie, Dongfa Liao, Xue Gou, Song Chen

**Affiliations:** 1 College of Medicine, Southwest Jiaotong University, Chengdu, Sichuan, China; 2 Department of Orthopaedics, The General Hospital of Western Theater Command, College of Medicine, Southwest Jiaotong University, Chengdu, Sichuan, China; 3 Institute of Biomedical Engineering, College of Medicine, Southwest Jiaotong University, Chengdu, China; 4 Pancreatic Injury and Repair Key Laboratory of Sichuan Province, The General Hospital of Western Theater Command, Chengdu, China

**Keywords:** mesenchymal stem cells, decellularized extracellular matrix (dECM), electrospun nanofiber scaffolds, osteogenesis, adipogenesis, chondrogenesis

There was a mistake in Figure 3E as published. Due to the multiple rounds of revision and the large number of panels, we inadvertently overlooked a mistake in Figure 3E during proofing: the images depicting the viability of IFPSCs and SDSCs on PDLLA were incorrectly presented. The corrected Figure 3 appears below.

**FIGURE 3:**
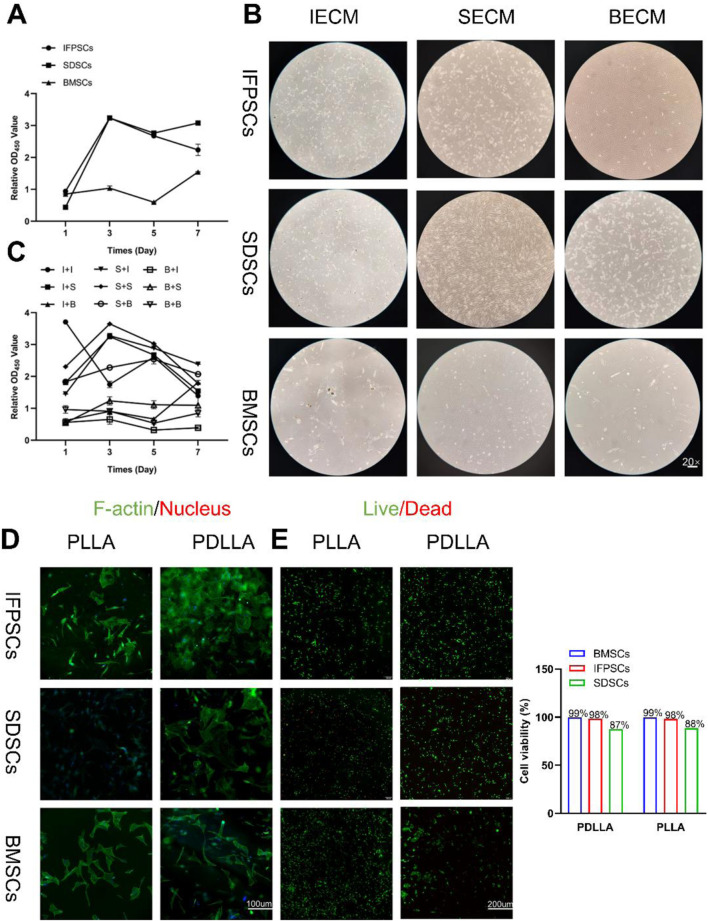
**(A)** Original stem cell proliferation rates were measured using a CCK-8 kit at 1, 3, 5, and 7 days. **(B)** Images of cells on dECM. **(C)** dECM pretreated-stem cell proliferation rates were measured using a CCK-8 kit at 1, 3, 5, and 7 days. (Key; I + I: IFPSCs + IECM (dECM deposited by IFPSCs); I + S: IFPSCs + SECM (dECM deposited by SDSCs); I + B: IFPSCs + BECM (dECM deposited by BMSCs); S + I: SDSCs + IECM; S + S: SDSCs + SECM; S + B: SDSCs + BECM; B + I: BMSCs + IECM; B + S: BMSCs + SECM; B + B: and BMSCs + BECM). **(D)** Cell adhesion morphology on PLLA and PDLLA nanofiber scaffolds. **(E)** Cell survival on electrospun nanofiber scaffolds made from PLLA and PDLLA. The cell viability was quantified using ImageJ software.

The original article has been updated.

